# Analysis of iris surface features in populations of diverse ancestry

**DOI:** 10.1098/rsos.150424

**Published:** 2016-01-13

**Authors:** Melissa Edwards, David Cha, S. Krithika, Monique Johnson, Esteban J. Parra

**Affiliations:** Department of Anthropology, University of Toronto Mississauga, Toronto, Ontario, Canada

**Keywords:** iris structure, Fuchs’ crypts, contraction furrows, Wolfflin nodules, pigment spots, conjunctival melanosis

## Abstract

There are many textural elements that can be found in the human eye, including Fuchs’ crypts, Wolfflin nodules, pigment spots, contraction furrows and conjunctival melanosis. Although iris surface features have been well-studied in populations of European ancestry, the worldwide distribution of these traits is poorly understood. In this paper, we develop a new method of characterizing iris features from photographs of the iris. We then apply this method to a diverse sample of East Asian, European and South Asian ancestry. All five iris features showed significant differences in frequency between the three populations, indicating that iris features are largely population dependent. Although none of the features were correlated with each other in the East and South Asian groups, Fuchs’ crypts were significantly correlated with contraction furrows and pigment spots and contraction furrows were significantly associated with pigment spots in the European group. The genetic marker *SEMA3A* rs10235789 was significantly associated with Fuchs’ crypt grade in the European, East Asian and South Asian samples and a borderline association between *TRAF3IP1* rs3739070 and contraction furrow grade was found in the European sample. The study of iris surface features in diverse populations may provide valuable information of forensic, biomedical and ophthalmological interest.

## 1. Introduction

The human iris is a complex tissue consisting of many different regions and strata. A healthy human eye typically has five different layers. The posterior-most layer is called the iris pigmented epithelium (IPE). This layer is tightly packed with cuboidal melanin-rich melanocytes in all healthy individuals and does not contribute significantly to variation in iris colour or structure [1–3]. Just above the IPE are two muscle layers, known as the sphincter muscle and the dilator muscle [1]. The two layers that are responsible for most of the variation between individuals are the anterior-border layer and the stromal layer. The anterior-border layer is made up primarily of fibroblasts and melanocytes [1,4,5]. By contrast, the stroma is a loose mesh of collagen fibres, melanocytes, fibroblasts and clump cells. The surface of the eye can also be divided into two regions: the pupillary zone and ciliary zone [4]. These regions are bounded by a ring of tissue known as the collarette, which is a product of the reabsorption of the pupillary membrane during development. There are differences in thickness between these two zones, which leads to variation in colour and structure [4].

**Figure 1.**
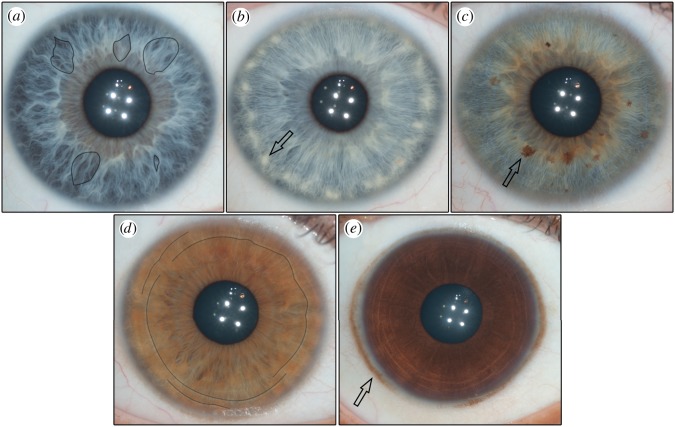
The five features found most commonly in the human iris. The arrows are pointing to the features in images b, c and e. Fuchs’ crypts (*a*) are lacunae in the anterior border of the iris which arise during resorption of the pupillary membrane. They may be either large or small and closely resemble windows. Four sample crypts are outlined in the image above. Wolfflin nodules (*b*) are small bundles of collagen that are the consequence of atrophy in the stromal layer of the iris. Pigment spots (*c*) are discrete areas of pigmentation that can be observed on the surface of the iris. Spots that distort the stromal layer are referred to as nevi and spots that do not distort the stromal layer are referred to as freckles. Contraction furrows (*d*) are rings that extend around the outer border of the iris. They closely resemble wrinkles and are the product of the contraction and dilation of the pupil. Furrows are typically discontinuous and staggered across the iris. In the above image, the black line follows the path of the furrows around the eye. Conjunctival melanosis (*e*) is spotting that can be observed on the scleral region surrounding the iris. It is usually benign, and is found more commonly in some ancestries than in others.

There are many textural elements that can be found in the healthy human eye. These include Fuchs’ crypts, Wolfflin nodules, pigment spots, contraction furrows and conjunctival melanosis (figure 1). Fuch’s crypts are diamond-shaped lacunae in the anterior-border layer of the iris, which first arise during the reabsorption of the pupillary membrane [6]. Wolfflin nodules are small bundles of collagen that accumulate along the outer edge of the iris [7,8]. Pigment spots are small regions of hyper-pigmentation in the anterior-border layer. They may be superficial (freckles) or distort the underlying stromal layer (nevi) [1,9,10]. Lastly, contraction furrows are folds that fall in rings around the outer edge of the iris [1]. They are believed to be the product of the dilation and the contraction of the pupil. Some irises may also show conjunctival melanosis, which is pigment spotting that can be found on the sclera surrounding the iris [11]. This tends to be more common in populations with darker irises.

Considerable research has been devoted to iris pigmentation variation [12–14]. However, very few studies have attempted to look at global variation in iris surface features. Although the functional consequences of these features remain largely unknown, they have become a topic of significant forensic, biomedical and ophthalmological interest. From a forensics perspective, a number of markers have been identified over the past 10 years that are capable of predicting pigmentation characteristics, such as hair, skin and iris colour, from crime scene DNA samples [15]. It has been suggested that some of the iris features, such as Wolfflin nodules and pigment spots, may have an influence on the perception of overall iris colour [12,16]. Therefore, a better understanding of the genetic basis of iris surface features may lead to improved eye colour predictor algorithms. Photographs of the iris may also represent a cost-effective alternative to more expensive ophthalmological procedures. Sidhartha *et al.* [17,18] recently characterized contraction furrow and Fuchs’ crypt grade in a Malaysian population living in Singapore. They found that both features were associated with iris thickness and/or the degree of iris angle closure. Thus, it may be possible to predict patient’s disease risk from iridial surface features. Lastly, although very little is known about the pathology of iris texture, there is some evidence that these features may influence individual health and well-being. In particular, nevi and pigment spots may be a risk marker for uveal melanoma [19,20].

As the textural elements in the iris have the potential to be of interest to many different disciplines, it has become imperative to develop a better understanding of the worldwide frequency and genetic basis of these traits. At present, iris features have been primarily studied in populations of European ancestry [21–23]. Very few studies have focused on non-European populations, and these studies have indicated that there are differences in the distribution of iris features between major population groups [24,25]. When iris texture was examined in Portuguese, Cape Verdean and Brazilian populations, for example, increasing European biogeographical ancestry was significantly associated with a greater number of pigment spots, Fuchs’ crypts and contraction furrows [24].

This paper has four primary goals: (i) to develop a method for describing iris surface features in populations of diverse ancestry; (ii) to characterize global differences in iris features across European, East Asian and South Asian populations; (iii) to look at correlations between the structural elements within each of the populations; and (iv) to look at the association between genetic markers that have been associated with iris surface features in European populations and our iris feature measurements.

## 2. Material and methods

### 2.1 Participant recruitment

Between 2012 and 2014, 1773 healthy volunteers of East Asian, European and South Asian ancestry were recruited at the University of Toronto to participate in a study looking at global pigmentation diversity. All participants ranged in age between 18 and 35 years, and were recruited using online and print advertisements that targeted members of the University of Toronto student community. A personal questionnaire asking about each participant’s maternal and paternal grandparents was administered in order to assess geographical ancestry. Individuals who stated that all of their grandparents came from Japan, Korea, China or Taiwan were categorized as East Asian, and individuals who stated that their grandparents originated in Bangladesh, Pakistan, Sri Lanka or India were categorized as South Asian. Participants were defined as European if their grandparents came from any region in Europe, other than Turkey. Only individuals who stated that all relatives came from the same general geographical region (i.e. East Asia) were included in the analysis. Admixed participants who came from two different regions (i.e. East Asian and Europe) were eliminated. When information about the grandparents was unknown, the self-reported ancestry of both parents was used to assess geographical ancestry. In total, our study included 623 participants of European ancestry, 475 of East Asian ancestry and 367 of South Asian ancestry. The remaining 308 participants were excluded from the analysis. In addition to determining ancestry, the personal questionnaire was also used to ensure that each participant was healthy, and had not been previously diagnosed with any ocular pigmentation-related disorders. Lastly, participants were asked to provide a self-assessment of their iris colour using the Fitzpatrick Phototype Scale [26].

### 2.2 Genotyping

A 2 ml saliva sample was taken from each participant using the Oragene+DNA (OG-500) collection kit (DNA Genotek, Canada). All participants were instructed not to eat, drink or smoke for at least 30 min prior to their appointment in order to ensure maximal sample purity. DNA was then isolated from each sample using the protocol provided by the manufacturer.

We selected four markers for genotyping that have either been directly associated with, or are purported to be associated with, iris texture in European populations [12,23]. These include *TRAF3IP1* rs3739070 (contraction furrows), *SEMA3A* rs10235789 (crypts), *DSCR9* rs7277820 (Wolfflin nodules) and *HERC1* rs11630290 (pigment spots).

All DNA samples were sent to LGC Genomics (USA) for genotyping. LGC Genomics uses a KASP-based genotyping method that combines allele-specific amplification with fluorescent resonance energy transfer technology. Twenty-nine samples were included as blind duplicates and 14 samples were included as blanks in order to check the quality of the genotyping results. The concordance rate for both blind duplicates and blanks was 100%.

### 2.3 Acquisition and processing of iris photographs

A photograph of each participant’s right iris was acquired using a Miles Research Professional Iris Camera (Miles Research, USA). This camera consists of a FujiFilm Finepix S3 Pro 12-Megapixel DSLR mounted on a 105-mm Nikkor macro lens. All photographs were taken in RAW format with an aperture of *f*19, a shutter speed of 1/125′ and an ISO of 200. A biometric coaxial cable was used to deliver light to the iris at a constant temperature in order to maintain colour and brightness fidelity and reduce the impact of ambient light. A Krypton 2.33 V light bulb was used as a focusing light, which allowed participants’ pupils to become adjusted to a standardized light source. A chin rest and camera mount was used to ensure that each photograph was taken from a standard distance and angle.

Photographs were converted to jpeg format and resized from 3043 by 2036 to 1200 by 803 pixels using Adobe Camera Raw in Adobe Photoshop CS5 (Adobe Systems Incorporated, USA). The white balance was set to flash, the contrast and blacks levels were set to zero and the default values were maintained for all remaining settings.

### 2.4 Iris analysis

**Figure 2.**
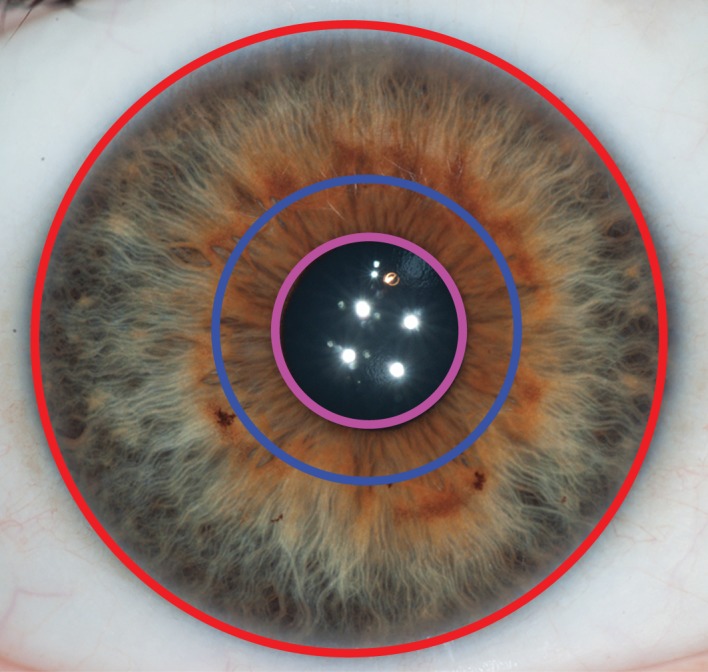
In steps 2–6 of the web-based application, the user draws a best fit circle around the pupillary ruff (purple circle), collarette (blue circle) and iris (red circle). This allows the program to identify the pupillary zone (bounded by the blue and purple circles) and the ciliary zone (bounded by the blue and red circles).

**Figure 3.**
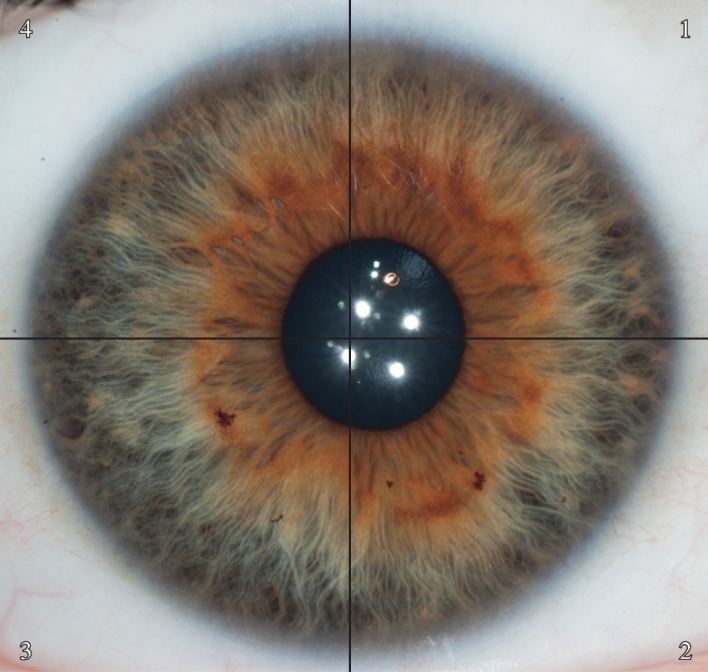
The program divides the iris into four different quadrants based on the user-defined centre of the iris. Quadrant 1 is the upper nasal quadrant, quadrant 2 is the lower nasal quadrant, quadrant 3 is the lower temporal quadrant and quadrant 4 is the upper temporal quadrant.

One of the authors (D.C.) designed a web-based application in order to accurately and reliably characterize iris texture. The web application can be accessed at http://iris.davidcha.ca/. Accounts can be set up for interested users by request. This program consists of 11 steps, which take approximately 2 min to complete for each iris. In the first step, the user is required to note whether or not the iris is significantly obstructed by eyelids, eyelashes or reflections (to the extent that any of the five structures of interest cannot be accurately characterized). Steps 2 to 6 have the user identify the approximate centre point of the iris, the approximate centre point of the pupil, and then draw a best fit circle around the scleral boundary, the collarette and the pupillary boundary. This allows the program to separate the iris into a ciliary zone and pupillary zone (figure 2), and divide the iris into four different quadrants based on the centre of the iris (figure 3).

In Step 7, the user must click on all of the pigment spots present in the iris. The iris is magnified by 1.5× times for this step, in order to more easily distinguish between pigment spots and other iridial textural elements. For each iris, the program records the number of pigment spots, as well as the quadrant in which each pigment spot is found.

In step 8, the user notes the presence and extension of contraction furrows. A rotating line, fixed at the centre point of the iris, is used to help the user determine whether or not the furrows cover more than 180° of the iris. In order to facilitate the identification of furrows, the iris is magnified by 1.5× for this step. The user also notes in which quadrants, if any, the furrows are found.

Wolfflin nodules are characterized in a manner similar to contraction furrows. In step 9, a rotating line is fixed at the centre point of the iris, and the user must determine whether nodules are present, and if so, whether or not they extend around more than 180° of the iris. As with contraction furrows, the user must also note in which quadrants the Wolfflin nodules can be found.

In step 10, the user clicks on the outermost edge of all of the crypts found in the iris. Crypts that originate from the collarette are characterized as either ‘small’ or ‘large’. Large crypts are defined as those that extend from the collarette into more than 50% of the ciliary zone. Small crypts are defined as those that do not extend into more than 50% of the ciliary zone. This is calculated automatically by the program. The program also records the quadrant in which each crypt is found. Crypts that do not originate from the collarette are manually defined as small. The iris is magnified by 1.5× for this step in order to increase the visibility of crypts in darker irises.

For the last step, the user must note whether or not there are any pigment rings or pigment spotting on the visible scleral region of the eye. The iris is obscured for this step, and only the sclera is visible.

After analysis of all 1465 irises, the program was used to output an Excel spreadsheet which contained the category for each of the five surface features, the quadrants in which these features were found, and the diameter of the iris in pixels. Detailed information about the position of each of the crypts and pigment spots identified could also be retrieved. All initial iris categorizations were carried out by M.E.

### 2.5 Characterization of iris surface features

**Figure 4.**
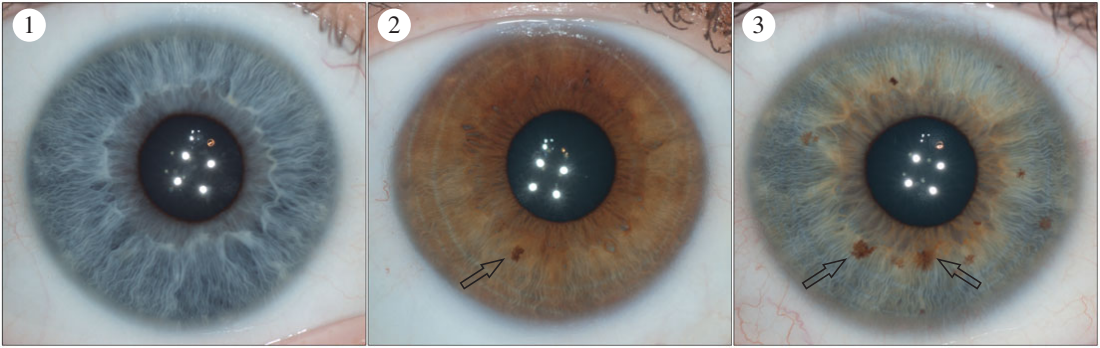
The pigment spot categories. Category 1: no pigment spots. Category 2: between 1 and 2 pigment spots. Category 3: more than two pigment spots. Any well-demarcated lesion that ranged in colour from tan to dark brown was considered a pigment spot. The black arrows illustrate example pigment spots.

**Figure 5.**
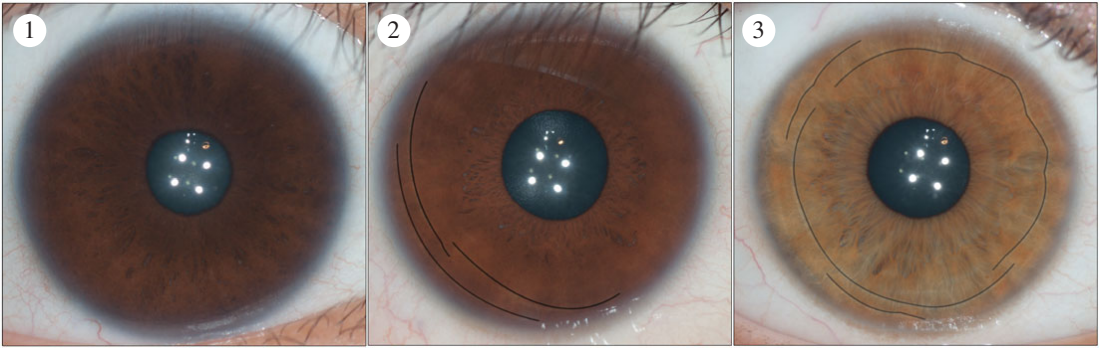
The contraction furrow categories. Category 1: no contraction furrows. Category 2: contraction furrows that extend less than 180° around the iris. Category 3: contraction furrows that extend more than 180° around the iris. The black lines follow the extension of contraction furrows around the iris.

**Figure 6.**
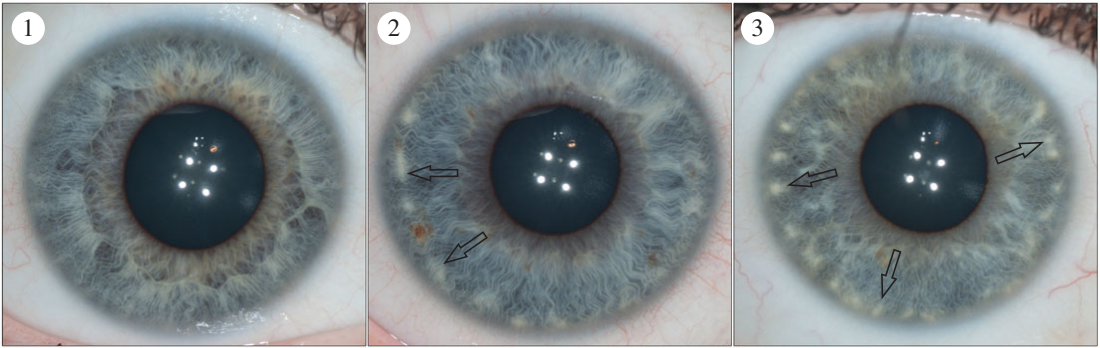
The Wolfflin nodule categories. Category 1: no Wolfflin nodules. Category 2: Wolfflin nodules that extend less than 180° around the iris. Category 3: Wolfflin nodules that extend more than 180° around the iris. Wolfflin nodules were defined as well-demarcated lesions that were white to orange in colour. The black arrows are pointing at example Wolfflin nodules.

**Figure 7.**
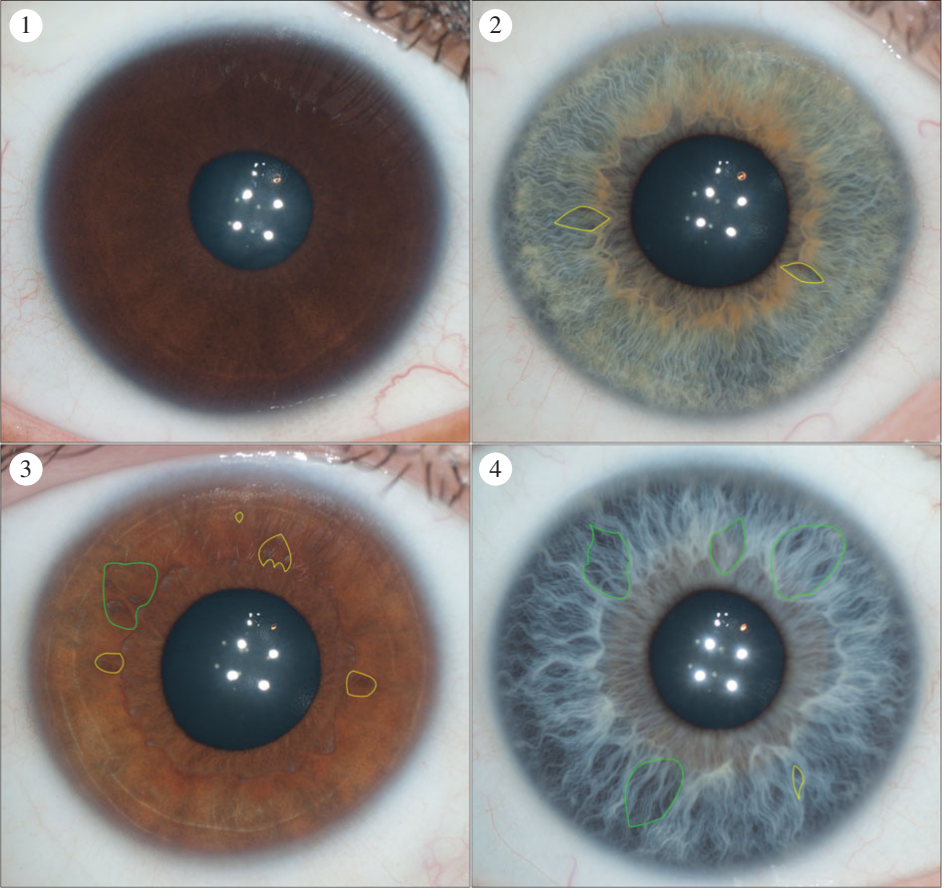
The crypt categories. Category 1: no crypts. Category 2: only small crypts centered around the collarette. Category 3: at least 1 large crypt located in fewer than three quadrants of the iris. Category 4: at least three large crypts located in three or more quadrants of the iris. Large crypts are defined as those that extend from the collarette into more than 50% of the ciliary zone. The web application used the scleral and iris boundaries to automatically distinguish between large crypts and small crypts. Examples of crypts are illustrated in the above images. Not all crypts are labelled in each image. Crypts that would be defined as large are bounded in green and crypts that would be defined as small are bounded in yellow.

**Figure 8.**
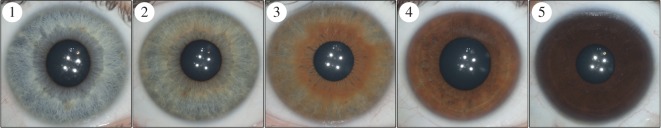
Self-described iris colour, as defined by the Fitzpatrick Phototype Scale. The Fitzpatrick scale characterizes iris colour across five categories: 1, light blue green or grey; 2, blue, green or grey; 3, hazel or light brown; 4, dark brown; and 5, brownish black. The photographs above represent a self-described example from each of the five categories.

**Figure 9.**
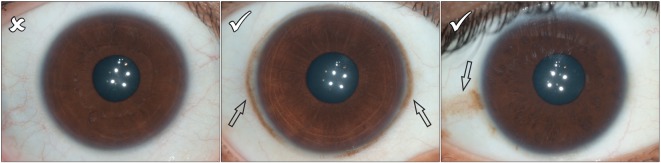
Conjunctival melanosis (black arrows) was measured using a presence or absence schema. Any iris that showed spotting on the sclera was characterized as having conjunctival melanosis. This spotting typically took the form of a ring around the iris, or as isolated spots on the sclera.

We developed iris feature categories that could capture variation in diverse populations with different frequencies of iris colour. These were primarily based on categorization systems that had been developed in prior studies [17,18,21–23]. However, they were modified to account for the pigmentation diversity present in our sample set. Pigment spots were initially measured using 4 grades: 1, no pigment spots; 2, between one and two pigment spots; 3, between three and five pigment spots; 4, more than five pigment spots. However, grades 3 and 4 were later collapsed into a single grade to account for the lower frequency of pigment spots in the East and South Asian groups compared to the European group (figure 4). As it is not possible to reliably differentiate between pigment spots and nevi in photographs, we chose pigment spot categories that attempted to capture the overall magnitude of spotting in the iris. Both contraction furrows and Wolfflin nodules were measured using a grading system that took into account the extension of these structures around the iris. Contraction furrows were measured across three grades: 1, no contraction furrows; 2, contraction furrows that extend less than 180° around the iris and 3, contraction furrows that extend more than 180° around the iris (figure 5). Wolfflin nodules were also measured using three grades: 1, no Wolfflin nodules; 2, Wolfflin nodules that extend less than 180° around the iris and 3, Wolfflin nodules that extend more than 180° around the iris (figure 6). Crypts were measured across a four grade system that attempted to capture the size and overall grade of crypts in the iris: 1, no crypts; 2, only small crypts cantered around the collarette; 3, at least one large crypt located in fewer than three quadrants of the iris and 4, at least three large crypts located in three or more quadrants of the iris (figure 7). Self-described iris colour was measured using the categories developed for the Fitzpatrick Phototype Scale (figure 8) [26]. These categories emphasize the intensity, rather than the shade, of the eye: 1, light blue, green or grey; 2, blue, green or grey; 3, hazel or light brown; 4, dark brown and 5, brownish black. Lastly, conjunctival melanosis was characterized using a presence or absence schema (figure 9).

### 2.6 Statistical analysis

Unless otherwise noted, statistical analyses were carried out using IBM Statistics SPSS (v. 20.0, SPSS Incorporated, USA). Correlations between the ordinal iris feature categories and iris colour were tested using Goodman and Kruskal’s gamma statistic, a measure of rank correlation typically used for ordinal traits. We report both the *G*-value and *p*-value for each of the correlations. Surface features were considered to be significantly correlated with each other or with iris colour if *p*<0.05. The relationships between iris feature and gender, age and iris width (the diameter of the best fit circle around the outer border of the iris) were examined using ordinal regression. The assumptions of proportional odds and goodness of fit were tested. Differences in iris feature frequency between the three sample sets were tested using the chi-square test. For features that were significantly different, additional pairwise comparisons between populations were conducted using an independent samples *t*-test with a Bonferonni correction for multiple comparisons. After correction, differences between samples were significant if *p*<0.0167. Differences in the width of the iris between the three populations were tested using a one-way ANOVA. Normality was tested using Q–Q plots and the Levene Statistic was checked before running the analysis.

Associations between the four polymorphisms of interest and the iris feature categories were evaluated using ordinal regression in each population, including other relevant covariates in the analyses (e.g. other iris structures, iris width or gender). The assumptions of proportional odds and goodness of fit were tested before running the analysis. Genotype deviations from Hardy–Weinberg proportions were evaluated using the Court Lab Calculator [27].

Four months after the initial iris classification, intra-rater reliability was assessed on 40 irises by M.E. and inter-rater reliability was assessed on 40 irises by D.C. using the linear weighted kappa statistic. This was done using the web application provided by GraphPad software (GraphPad, USA).

## 3. Results

A total of 475 East Asian, 623 European and 367 South Asian irises were evaluated using the iris structure program (table 1). Of these, 14 irises (eight East Asian, four European and two South Asian) were judged to be too obscured or blurry to accurately characterize, and were excluded from the analysis. One additional participant of South Asian ancestry with a pigmentation-related ocular disorder (albinism) was also removed from the study. None of the remaining participants reported ocular disorders. However, as medical history was self-reported, it is possible that additional participants with ocular disorders may have been incorporated into the study. The intra- and inter-rater reliability was assessed using the kappa statistic (for additional information, see Material and Methods section) [28]. The intra- and inter-rater reliabilities were good for all five structures (intra-rater kappa values; crypt *κ*=0.881, furrow *κ*=0.835, nodule *κ*=0.884, pigment spot *κ*=0.889, melanosis *κ*=0.827; inter-rater kappa values; crypt *κ*=0.809, furrow *κ*=0.857, nodule *κ*=1.000, pigment spot *κ*=0.898, melanosis *κ*=0.867).

**Table 1.** General descriptive statistics for the East Asian, European and South Asian irises.

population	irises included	females	males	average age	average iris width
East Asian	467	320	147	21.61	376.72
European	619	376	243	22.65	394.45
South Asian	364	246	118	20.67	384.92

The width of the iris was significantly different across the three sample sets (*F*=200.161, *p*<0.001), with East Asians having the smallest iris widths (*mean*=376.72 pixels), followed by South Asians (*mean*=384.92) and then Europeans (*mean*=394.45 pixels).

Within the European sample set, there was a significant negative correlation between the grade of Fuchs’ crypts and the extension of contraction furrows (*G*=−0.474, *p*<0.001) and a significant, but weaker, negative correlation between the grade of Fuchs’ crypts and the number of pigment spots (*G*=−0.238, *p*<0.001). There was also a significant, positive correlation between the extension of furrows and the number of pigment spots (*G*=0.218, *p*=0.007). No significant correlations between the five iridial structures were found in either the South or East Asian sample sets. In the European sample set, self-reported darker iris colour showed a significant positive correlation with the extension of contraction furrows (*G*=0.461, *p*<0.001) and a significant negative correlation with Wolfflin nodules (*G*=−0.409, *p*<0.001). In the South Asian sample set, in contrast with what was observed in the European sample set, darker iris colour showed a significant negative correlation with the extension of contraction furrows (*G*=−0.233, *p*=0.041).

Gender was significantly associated with crypt grade in the East Asian (Nagelkerke *R*^2^=0.013, *p*=0.019), European (Nagelkerke *R*^2^=0.022, *p*<0.001) and South Asian (Nagelkerke *R*^2^=0.037, *p*<0.001) samples. In all three groups, males had a significantly higher crypt grade than females (East Asian male/female OR=1.54, European male/female OR=1.72, South Asian male/female OR=2.07). Gender was not associated with any of the other four iridial structures. Iris diameter was significantly associated with a higher number of pigment spots in the European (Nagelkerke *R*^2^=0.012, *p*=0.009) and South Asian sample sets (Nagelkerke *R*^2^=0.018, *p*=0.041). Iris diameter was also significantly associated with more extended Wolfflin nodules in Europeans (Nagelkerke *R*^2^=0.021, *p*=0.001). No associations were found between age and iris structure in any of the three groups.

All five structures examined showed significant differences in frequency between the three sample sets (Fuchs’ crypts: *χ*^2^=67.388, *p*<0.001; contraction furrows: *χ*^2^=186.819, *p*<0.001; pigment spots: *χ*^2^=260.587, *p*<0.001; Wolfflin nodules: *χ*^2^=350.627, *p*<0.001; conjunctival melanosis: *χ*^2^=273.177, *p*<0.001). Additional pairwise independent sample *T*-tests with a Bonferonni correction were run in order to determine the source of these differences. Europeans had a significantly higher grade of Fuchs’ crypts (*T*=8.333, *p*<0.001), more extended contraction furrows (*T*=10.802, *p*<0.001), more pigment spots (*T*=14.686, *p*<0.001) and more extended Wolfflin nodules (*T*=18.028, *p*<0.001) than individuals of East Asian ancestry and a significantly higher grade of crypts (*T*=2.961, *p*=0.003), greater number of pigment spots (*T*=15.571, *p*<0.000) and more extended Wolfflin nodules (*T*=16.345, *p*<0.001) than individuals of South Asian ancestry. The South Asian and East Asian samples had a higher proportion of individuals with conjunctival melanosis than the European sample (South Asian: *T*=15.954, *p*<0.001; East Asian: *T*=6.674, *p*<0.001). There was no significant difference in contraction furrow extension between the European and South Asian groups (*T*=−0.306, *p*=0.759). Individuals of South Asian ancestry had a significantly higher grade of crypts (*T*=4.231, *p*<0.001), more extended contraction furrows (*T*=10.474, *p*<0.001) and a higher frequency of conjunctival melanosis (*T*=6.674, *p*<0.001) than individuals of East Asian ancestry. However, there was no significant difference in the number of pigment spots (*T*=−1.527, *p*=0.127) and the presence of Wolfflin nodules (*T*=2.372, *p*=0.018) between these two groups. Self-described iris colour was significantly different (*χ*^2^=892.674, *p*<0.001) across all three samples. Individuals of East Asian ancestry had significantly darker eyes than individuals of South Asian (*T*=4.327, *p*<0.001) and European (*T*=40.776, *p*<0.001) ancestry and individuals of South Asian ancestry had significantly darker eyes (*T*=32.724, *p*<0.001) than individuals of European ancestry.

Genotype proportions were in agreement with the expected Hardy–Weinberg proportions, with the exception of moderate deviations observed for *DSCR9* rs7277820 in the European (*χ*^2^=5.373, *p*=0.020) and South Asian (*χ*^2^=3.930, *p*=0.047) sample sets and *HERC1* rs11630290 in the South Asian sample set (*χ*^2^=5.224, *p*=0.022). We looked at the association between *SEMA3A* rs10235789 and Fuchs’ crypts, *TRAF3IP1* rs3739070 and contraction furrows, *DSCR9* rs7277820 and Wolfflin nodules, and *HERC1* rs11630290 and pigment spots. In the statistical tests, we included as covariates the variables that were significantly associated with each iris structure in our exploratory analyses (e.g. other iris structures, iris width, self-reported iris colour and gender).

Fuchs’ crypt grade was significantly associated with *SEMA3A* rs10235789 in all three sample sets (table 2). In the European sample set, having one copy of the derived C allele significantly increased the odds of having a higher grade of crypts by 1.507 (*p*=0.023) and having two copies of the derived C allele significantly increased the odds of having a higher grade of crypts by 2.203 ( *p*<0.001). In the South Asian sample, having one copy of the derived C allele significantly increased the odds of having a higher grade of crypts by 2.206 (*p*<0.001) and having two copies of the derived C allele significantly increased the odds of having a higher grade of crypts by 2.721 (*p*=0.011). In the East Asian sample set, having one copy of the derived C allele significantly increased the odds of having a higher grade of crypts by 1.679 ( *p*=0.018). As there were fewer than five individuals with two copies of the derived C allele, they were eliminated from this analysis.

**Table 2.** The results of the genetic ordinal regression. Significance values and odds ratios for each genotype are presented for each population. Genotypes labelled with an ‘a’ refer to the ancestral genotype.

polymorphism	population	genotypes	significance	odds ratios
rs10235789 (Fuchs’ crypts)	East Asian	CC	—	—
		CT	0.018	1.679
		TT	a	a
	European	CC	<0.001	2.203
		CT	0.023	1.507
		TT	a	a
	South Asian	CC	0.011	2.721
		CT	<0.001	2.206
		TT	a	a
rs3739070 (contraction furrows)	East Asian	AA	—	—
		CA	0.956	0.980
		CC	a	a
	European	AA	0.051	6.385
		CA	0.333	2.601
		CC	a	a
	South Asian	AA	—	—
		CA	0.168	0.360
		CC	a	a
rs11630290 (pigment spots)	East Asian	TT	—	—
		CT	0.173	0.241
		CC	a	a
	European	TT	0.187	1.685
		CT	0.998	1.000
		CC	a	a
	South Asian	TT	—	—
		CT	0.136	0.536
		CC	a	a
rs7277820 (Wolfflin nodules)	European	AA	0.375	0.758
		GA	0.345	0.794
		GG	a	a

The association between contraction furrow grade and *TRAF3IP1* rs3739070 was borderline significant in the European group but not in the East and South Asian sample sets (table 2). Having two copies of the derived A allele increased the odds of having more extended contraction furrows by 6.385 (*p*=0.051). However, having only one copy of the A allele did not significantly (*p*=0.333) increase the odds. Both pigment spot grade and Wolfflin nodule grade were not significantly associated with their respective markers in any of the three groups examined.

## 4. Discussion

The use of a program to characterize iris surface features has a number of advantages over traditional qualitative assessments of iris photographs. For one, it allows for the storage and retrieval of a substantial amount of information that would not be easy to obtain using descriptive methods. This includes the size of the iris, the position in which each pigment spot and crypt is found, and the distribution of traits across the different quadrants of the iris. In addition, the program displays irises in a random order to limit bias, can handle large amounts of data and is relatively fast. It is also simple to set up replicate users for intra- and inter-reliability estimates. The inter- and intra-rater reliability estimates were both good for all five iris structures examined, which underscores the ability of the program to return consistent and repeatable results.

The iris structure categories that we developed were designed to account for the extensive iris texture variation that is inherent in global populations. Previous attempts to study iris structure have primarily been targeted at single, more homogeneous populations [17,18,21–23]. In this paper, we have attempted to expand on these methods and develop categories that can capture iris structure variation in global populations with different iris characteristics.

### 4.1 Fuchs’ crypts

Substantial amounts of hypoplasia can develop in the iris following the absorption of the pupillary membrane. The optic vessels first appear around the fourth week of fetal life, and the anterior layer begins to grow in front of the lens around the second embryonic month [1,4,29]. This ultimately forms the iridopupillary membrane, which covers the entire iris. By the sixth month, this membrane begins to atrophy and is gradually reabsorbed until the pupil is completely free of mesodermal tissue. It has been suggested that the anterior layer that remains is a rudimentary tissue that has no major function in humans [6]. As a result, there is a sparsity of tissue in this layer. Most irises have a moderate amount of hypoplasia around the collarette. However, in some eyes, this hypoplasia can extend widely into the ciliary zone. This leads to the formation of diamond-shaped lacunae known as Fuchs’ crypts. These lacunae are primarily believed to be a phylogenetic defect that results from the decreasing importance of the anterior layer over evolutionary history [6]. However, secondary lacunae can also gradually form due to the pushing and pulling effects of the pupil on the anterior layer when it dilates and contracts. Thus, it is not surprising that age has been associated with a higher number of crypts in prior studies [22]. For Fuchs’ crypts, we attempted to develop a categorical system that could capture the total amount of hypoplasia in the iris. Irises that contained very little hypoplasia, or hypoplasia cantered solely along the collarette, were placed into the first two categories. Irises that had a greater amount of hypoplasia that extended into at least 50% of the ciliary zone were placed in the latter two categories.

In all three sample sets, the largest number of participants fell into the second category (small crypts around the collarette in at least three quadrants of the iris). However, individuals of European descent had a greater probability of having a higher crypt grade than individuals of South Asian descent, and individuals of South Asian ancestry had a greater probability of having a higher crypt grade than individuals of East Asian descent (table 3). Among East Asians, only 29.8% of participants had crypts that extended into the ciliary zone, compared with 52.9% of Europeans and 43.4% of South Asians. There may be several explanations for this finding. Individuals of East Asian ancestry have significantly darker irises than those of South Asian ancestry or European ancestry. It is possible that the presence of greater amounts of melanin in the anterior stromal layer may make the iris less prone to the development of both primary and secondary crypts. However, this is unlikely as the frequency of crypts appears to be independent of iris colour within populations [22]. Iris width may be another explanation, as we noted that individuals of East Asian ancestry had significantly smaller irises than those of South Asian and European ancestry. However, we were unable to find an association between iris width and crypt grade within populations. As the frequency of Fuchs’ crypts is believed to be associated with the overall stability of the anterior-border layer, it is possible that there are other genetic and developmental population differences that affect the stability of this layer.

**Table 3.** The percentage (and number) of individuals with each category of Fuchs’ crypts, contraction furrows pigment spots, Wolfflin nodules, melanosis and iris colour in all three populations.

trait	category	East Asian	European	South Asian
Fuchs’ crypts	0	20.3% (95)	9.2% (57)	15.1% (55)
	1	49.9% (233)	38.0% (235)	41.5% (151)
	2	19.7% (92)	30.9% (191)	25.0% (91)
	3	10.1% (47)	22.0% (136)	18.4% (67)
contraction furrows	0	15.2% (71)	6.6% (41)	3.8% (14)
	1	37.7% (176)	11.1% (69)	15.4% (56)
	2	47.1% (220)	82.2% (509)	80.8% (294)
pigment spots	0	77.9% (364)	42.1% (260)	83.2% (303)
	1	20.6% (96)	38.4% (238)	14.8% (54)
	2	1.5% (7)	19.5% (121)	1.9% (7)
Wolfflin nodules	0	100% (467)	62.7% (388)	98.6% (359)
	1	0% (0)	12.0% (74)	0.5% (2)
	2	0% (0)	25.4% (157)	0.8% (3)
melanosis	0	76.7% (358)	97.9% (606)	54.9% (200)
	1	23.3% (109)	2.1% (13)	45.1% (164)
eye colour	0	0% (0)	11.8% (73)	0% (0)
	1	0% (0)	45.1% (279)	1.6% (6)
	2	4.5% (21)	23.1% (143)	8.2% (30)
	3	45.4% (212)	19.1% (118)	51.9% (189)
	4	50.1% (234)	1.0% (6)	38.2% (139)

In all three groups, gender was weakly associated with Fuchs’ crypts. Most notably, males consistently had greater odds of having a higher crypt grade than females. We were not able to replicate an association between Fuchs’ crypts and age [22]. However, this was most likely because of the relatively narrow age range used in our study, compared with prior studies where participants ranged in age from children to seniors.

*SEMA3A* rs10235789 is one marker that appears to be associated with variation in Fuchs’ crypts [23]. This marker, which is believed to play a role in the initial development of the pupillary membrane, has previously been associated with approximately 1.5% of the variation in Fuchs’ crypts in an Australian sample of European ancestry. When we looked at the association between *SEMA3A* rs10235789 and Fuchs’ crypt grade in our European, East Asian and South Asian samples, we found significant associations in all three groups. The effects of *SEMA3A* rs10235789 appear to be additive, with two copies of the derived C allele having a greater effect than one copy. The geographical distribution of the *SEMA3A* rs10235789 polymorphism may help to explain some of the differences observed in the frequency of crypts in the three groups. The derived C allele is found at a very high frequency in our European sample (0.48), while it is found at much lower frequencies in the East Asian (0.08) and South Asian (0.28) groups. Therefore, we would expect crypts to be found at a lower frequency in the latter groups. Given the role of *SEMA3A* rs10235789 in the absorption of the pupillary membrane, it is possible that there are population differences in the stability of the anterior-border layer after pupil reabsorption in the different population groups.

### 4.2 Pigment spots

Pigment spots are discrete areas of dark pigmentation that are found on the anterior-border layer of the iris [9]. There are many different types of spots, the most common of which are iris freckles and nevi. Both variants of pigment spots are similar topographically, but are very different ultrastructurally [1,10]. Freckles, which range from light to dark in colour, are found in 50–60% of healthy adults [9,30]. They lie flat on the surface of the iris, and do not distort the iris stroma. By contrast, nevi are found only in 4–6% of adults, and look like well-demarcated nodular lesions [9]. They tend to be more common on the lower half of the iris and may increase in size over time [19]. Unlike freckles, nevi do distort the underlying stromal layer. Although older individuals have a predisposition for both types of pigment spots, they may be found in all ages [21,30].

Pigment spotting was significantly more common in individuals of European ancestry than in individuals of East or South Asian ancestry (table 3). Over half of our European sample (57.9%) showed some degree of iridial spotting, compared with only 22.1% of East Asians and 16.7% of South Asians. This finding is not surprising. As pigment spots are discrete regions of brown or black pigment, we would expect to see fewer spots in populations with a predisposition for darker iris colours given that regions of hyper-melanin can be difficult to distinguish in darker eyes. Interestingly, in all three groups, pigment spots were most likely to be found in the lower temporal quadrant of the iris (table 4). Several prior studies have noted that pigment spots preferentially appear on the lower half of the iris [31]. This is because sun exposure appears to be a risk factor for the development of iridial spots, and the lower half of the iris is least protected by the eyelids. However, we only noted this preference on the lower temporal quadrant, not on the lower nasal quadrant. In fact, the second most common quadrant for pigment spotting was the upper temporal quadrant. It is possible that the upper temporal quadrant may be less protected by the eyelids than the lower nasal quadrant, leading to this discrepancy.

**Table 4.** The percentage (and number) of irises with large crypts, contraction furrows, pigment spots and Wolfflin nodules in each of the four quadrants of the iris.

trait	population	quadrant 1	quadrant 2	quadrant 3	quadrant 4
large crypts	East Asian	10.9% (51)	15.8% (74)	14.3% (67)	18.4% (86)
	European	31.5% (195)	35.5% (220)	25.8% (160)	29.2% (181)
	South Asian	23.6% (86)	29.9% (109)	22.5% (82)	22.3% (81)
furrows	East Asian	26.8% (125)	70.4% (329)	82.4% (385)	61.0% (285)
	European	82.1% (508)	84.8% (525)	89.2% (552)	84.3% (522)
	South Asian	70.6% (257)	91.2% (332)	95.1% (346)	84.1% (306)
pigment spots	East Asian	2.8% (13)	5.1% (24)	10.3% (48)	8.1% (38)
	European	17.3% (107)	18.6% (115)	31.7% (196)	31.2% (193)
	South Asian	2.7% (10)	4.9% (18)	6.3% (23)	6.6% (24)
nodules	East Asian	0% (0)	0% (0)	0% (0)	0% (0)
	European	23.7% (147)	28.6% (177)	34.9% (216)	31.2% (193)
	South Asian	0.8% (3)	1.1% (4)	1.4% (5)	1.4% (5)

We did not find a correlation between pigment spotting and gender or age in any of the three groups. However, we did note an association between iris width and pigment spot grade in the European and South Asian sample sets. Given the well-established link between sun exposure and iris pigment spots, it may simply be the case that a wider iris has more contact with the sun than a smaller iris. We were also not able to identify an association between *HERC1* rs11630290 and pigment spots in any of the three samples. This is not surprising, as *HERC1* rs11630290 was the weakest association reported in prior studies. It is possible that our sample size was too small to replicate this association.

### 4.3 Contraction furrows

Contraction furrows, which are produced by the contraction and dilation of the pupil, are deep depressions that lie around the outer periphery of the iris [1,30]. Although each contraction furrow rarely extends more than an iris quadrant in length, there are typically many furrows staggered around the eye. It has been suggested that the overall thickness and density of the iris play an important role in their formation and overall appearance [22,23]. Although prior studies have used several different systems for describing contraction furrows, we were primarily interested in looking at their overall extension around the iris. This is because furrow extension is easier to quantify than furrow depth or number of furrows when looking at irises of varying shades.

In all three samples, there were very few individuals with the lowest grade of contraction furrows (15.2% of East Asians, 6.6% of Europeans, 3.8% of South Asians). Individuals of South Asian and European ancestry had similar distributions of contraction furrow grade (table 3). However, participants of East Asian ancestry had a significantly lower furrow grade than either South Asians or Europeans. In several prior studies, contraction furrow grade has been associated with both darker irises and a thicker peripheral iris within populations [17,22,24]. Thus, it is unexpected to see an overall lower grade of contraction furrows in the East Asian sample, given that as this group has the darkest self-described eye colour and the fact that East Asian individuals have also been found to have a thicker peripheral iris than individuals of European ancestry [32]. This suggests that some factor other than iris colour or iris depth is responsible for generating differences in contraction furrow grade between populations. One explanation may be iris width and iris area. There is some evidence that a higher overall iris area (irrespective of iris depth) may be associated with a higher grade of contraction furrows [17]. In our sample, individuals of East Asian ancestry had a significantly smaller iris width than individuals of European or South Asian ancestry. Likewise, prior studies have found that individuals of East Asian ancestry have an overall smaller iris area than individuals of other population groups [33]. Although we were unable to find a significant association between contraction furrow grade and iris width in any of the population groups, this association was close to the established significance value of *p*<0.05 in the East Asian (*p*=0.096) and European (*p*=0.073) groups.

*TRAF3IP1* rs3739070 has been associated with approximately 1.7% of the variation in contraction furrows in Australian individuals of European ancestry [23]. It has been suggested that this marker may play a role in determining the overall thickness and density of the iris. We were able to detect a borderline significant association (*p*=0.051) between *TRAF3IP1* rs3739070 and contraction furrows in our European sample. The derived allele appears to have a recessive effect, with one copy of the derived allele not increasing the odds of having more extended contraction furrows. We estimated that having two copies of the derived A allele increases the odds by 6.385. However, it is important to note that we only had five individuals who were homozygous for the ancestral allele. In both East and South Asians, the ancestral *TRAF3IP1* rs3739070 allele also has a very low frequency. No participants of South Asian descent, and only one individual of East Asian descent, were homozygous for the ancestral A allele. In addition, the frequency of heterozygotes was also very low in both populations. Thus, it is not surprising that we were unable to find an association between *TRAF3IP1* and furrow grade in either group. It is likely that markers other than *TRAF3IP1* rs3739070 play a role in the development of contraction furrows in populations of non-European ancestry. East Asian populations may be an ideal group for studying the genetic basis of contraction furrows. Unlike the European and South Asian samples, where there is little trait variation and the vast majority of individuals have highly extended furrows, participants of East Asian ancestry were more likely to fall into the lower furrow grades.

### 4.4 Wolfflin nodules

Wolfflin nodules are small (0.1–0.2 mm) circular lesions that are distributed uniformly along the outer border of the ciliary zone [7,21]. They primarily comprise atrophied collagen from the anterior-border and stromal layers. Wolfflin nodules are highly associated with iris colour, and are much more common in light-eyed individuals than in dark-eyed individuals. For example, in one study of 123 brown-eyed East Asian children, Wolfflin nodules were absent from all participants [34]. Much attention has been devoted to Wolfflin nodules during the past 50 years due to their structural similarity to Brushfield spots, an iris feature found in 85–90% of Down’s syndrome patients [7,35]. Although the genetic basis of Brushfield spots remains largely unknown, they closely resemble Wolfflin nodules and also appear to be restricted to individuals with light-coloured eyes. However, they tend to be larger, less uniform, and located closer to the mid-zone of the iris.

We decided to characterize Wolfflin nodules based on their overall extension around the iris. Individuals who had Wolfflin nodules that extended more than 180° around the iris were put into the highest grade, and individuals who had Wolfflin nodules that extended less than 180° were placed in the second grade. Individuals with no discernable Wolfflin nodules were placed in the lowest grade. Interestingly, very few individuals fell into the second category and participants were more likely to have no Wolfflin nodules or Wolfflin nodules that extended around the entire iris (table 3).

Unsurprisingly, Wolfflin nodules were significantly more common in the European sample, with 25.4% of this group having Wolfflin nodules that extended more than 180° around the iris, compared with 0% of East Asians and 0.8% of South Asians. Given the strong association between Wolfflin nodules and iris colour, we would expect to see fewer Wolfflin nodules in the primarily brown-eyed East and South Asian populations. It has been suggested that both Wolfflin nodules and Brushfield spots are not actually absent in brown irises, but merely obscured by the abundance of melanin particles in the anterior-border layer [36]. Therefore, it is possible that methods other than colour photography may be necessary to accurately characterize Wolfflin nodules in populations of diverse ancestry. Interestingly, we did find an association between larger iris width and more extended Wolfflin nodules in the European population. As Wolfflin nodules are composed of atrophied collagen from the stromal layer, it is possible that larger irises may be more prone to the accumulation of nodules in the anterior stromal layer.

In a recent genome-wide association study on iris colour in a population of Danish ancestry, *DSCR9* rs7277820 was found to be associated with iris colour variation [12]. It was suggested that this marker may actually be correlated with Wolfflin nodules, due to its presence in the Down’s syndrome Critical region. When we looked at the association between *DSCR9* rs7277820 and Wolfflin nodules in the European sample, we were not able to find a significant relationship.

### 4.5 Conjunctival melanosis

Finally, the last trait that we looked at was conjunctival melanosis. In some irises, there are areas of pigment spotting on the scleral region surrounding the iris [11]. These spots can comprise either of discrete regions, or rings surrounding the iris. Conjunctival melanosis has not yet been widely explored. However, it does appear to be found more commonly in populations with darker irises. This was largely reflected in our sample. Here, almost half of our South Asian participants showed some form of conjunctival melanosis, compared to only 23.3% of East Asians and 2.1% of Europeans (table 3). It is important to note, however, that these results may be an underestimate given that the upper and lower portions of the sclera were not fully visible in our sample. Interestingly, the presence of conjunctival melanosis does not appear to be entirely linked to iris colour, as the distribution of this trait is very different among the East and South Asian participants.

### 4.6 Correlations of iris features

Correlations between iris features and gender, age and iris width were examined in all three population groups. Within the European population we were able to replicate a number of the correlations that have been identified in prior studies. These include: (i) a higher grade of crypts is correlated with less extended contraction furrows [22]; (ii) a higher grade of crypts is correlated with fewer pigment spots [24]; and (iii) more extended contraction furrows are correlated with a greater amount of pigment spotting [22]. We were not initially able to replicate the association between crypt grade and Wolfflin nodule grade [22]. However, we were interested in determining if this was just because of the higher frequency of brown-eyed Europeans in our sample, in comparison with the previous studies reporting the correlation. When we restricted our sample to individuals who self-described their iris colour as either ‘light blue, green or grey’ or ‘blue, green or grey’, a higher grade of crypts (*p*<0.044, *G*=0.142) and more extended contraction furrows (*p*=0.001, *G*=0.343) were both correlated with more extended Wolfflin nodules. This emphasizes the effects that iris colour can have on the study of global Wolfflin nodule variation. In contrast with the significant correlations observed in the European sample, none of the five traits were significantly correlated in either the East or South Asian populations. There are several potential explanations for this: (i) iris colour may be obscuring some of the associations; (ii) there may be population differences responsible for the lack of correlations; or (iii) our ability to identify significant correlations in the East Asian and South Asian samples was hampered due to smaller sample sizes.

### 4.7 Future research

Several prior studies have suggested that the distribution of iris features may be population dependent [24,25]. In this paper, we looked at the global distribution of Fuchs’ crypts, Wolfflin nodules, contraction furrows, pigment spots and conjunctival melanosis in participants of East Asian, European and South Asian ancestry. We found that all five traits showed significant differences in frequency across the three groups. We also showed that *SEMA3A* rs10235789 is significantly associated with crypts not only in the European sample, but also in the East Asian and South Asian samples. By contrast, *TRAF3IP1* rs3739070 is only correlated with contraction furrows in Europeans. Lastly, we were able to replicate all of the iris feature correlations that had been identified in prior studies in our European sample. However, we were not able to identify any correlations in the East and South Asian groups. Future research will be necessary to explore the frequency of these features in other populations, such as African and Hispanic groups.

There are still many gaps in our understanding of iris surface features. At present, very little is known about the genetic basis and global distribution of Wolfflin nodules. This is primarily because it is difficult to study this trait in populations that are primarily composed of individuals with brown eyes. While it was initially suggested that these collagen bundles were absent in darker irises, it has since been established that they are most likely masked when there are large amounts of melanin particles in the anterior stromal layer. At present, the only way to accurately characterize Wolfflin nodules is through magnification [36]. Pigment spots are similarly very difficult to study. Most notably, we do not have a way of confidently differentiating between pigment freckles and nevi from photographs of the iris. Nevi and freckles both appear to have a different relationship with the development of ocular disorders such as uveal melanoma [19,20]. Therefore, it would be extremely useful to develop ways of distinguishing these features in photographs.

We suggest that infrared photography may provide a potential solution to these issues. As infrared light is neither absorbed nor reflected by melanin, it may provide a way of visualizing traits, such as Wolfflin nodules, that lie below the melanin granules in the anterior-border layer. In addition, as the primary difference between nevi and freckles is whether or not the underlying stromal layer is distorted, infrared photography may provide a means of identifying the spots that have an effect on this layer. The field of iris recognition already relies heavily on the use of infrared photography. However, it also amalgamates all of the structures in the iris into a single code [37]. Looking at infrared photographs from a qualitative perspective may provide valuable information about the distribution of both pigment spots and Wolfflin nodules in global populations.

Iris features are beginning to play an increasingly important role in many different fields. However, there are still many gaps in our knowledge of these traits. Future research will be necessary in order to determine the functional differences in these traits in global populations, as well as the effects that these traits may have on population-specific ocular diseases and disorders.

## Data accessibility

The iris structure program is a web application that is available at http://iris.davidcha.ca/. Accounts can be set up by request.
